# The role of actin cytoskeleton component Arp2/3 in inflammatory response

**DOI:** 10.3389/fcell.2025.1631187

**Published:** 2025-11-17

**Authors:** Jianxiao Xing, Ying Wang, Yanyang Liang, Jiao Li, Yuanjun Yao, Junqin Li, Kaiming Zhang

**Affiliations:** Shanxi Key Laboratory of Stem Cells for Immunological Dermatosis, State Key Breeding Laboratory of Stem Cells for Immunological Dermatosis, Taiyuan Central Hospital, Taiyuan, China

**Keywords:** actin cytoskeleton, inflammation, ARP2/3, cell migration, WASP

## Abstract

Actin regulatory protein plays an important role in immune-related diseases and affects cellular behavior by regulating the dynamic changes of the cytoskeleton. This regulation is crucial for maintaining the fine balance in the body’s biological processes, and can effectively prevent and control the occurrence and development of acute or chronic inflammation, thereby avoiding the appearance of various diseases. The Arp2/3 complex, an evolutionarily conserved molecular machinery, regulates actin cytoskeleton dynamics and nucleates branched actin networks. Upon activation, the Arp2/3 complex binds to the lateral face of pre-existing actin filaments and nucleates daughter filament assembly, generating branched actin networks through this Y-junction formation mechanism. However, the research on how actin is involved in regulating the inflammatory process has only gradually become clear recently. The article mainly summarizes the functions of the actin cytoskeleton, the role of the Arp2/3 complex, and its specific functions in the inflammatory response.

## 1 Introduction

The cytoskeleton is a key regulator of the structure and function of eukaryotic cells. It consists of a variety of filamentous structures formed by the self-assembly and polymerization of specialized proteins (Hohmann and Dehghani, 2019), with three core components: actin filaments, intermediate filaments, and microtubules. These cytoskeletal components play a crucial role in regulating the function of inflammatory cells (Ridge et al., 2022); collectively, they mediate essential cellular processes, including cell division, migration, adhesion, cell-to-cell interactions, as well as actin cleavage, assembly, and remodeling (Mostowy and Cossart, 2012; Tang and Gerlach, 2017).​

Notably, defects in the assembly and remodeling of cytoskeletal components are central drivers of the development and progression of numerous diseases. For instance, microtubules not only regulate pathogen sensing via inflammatory factors, immune synapse assembly, and vascular leakage in tissues (Loeven et al., 2020; Martin-Cofreces et al., 2014; Karki and Birukova, 2017), but also mediate multiple inflammation-related pathways—such as cell adhesion and neutrophil recruitment, superoxide production, inflammasome activation, and the nuclear factor kappa B (NF-κB) pathway induced by tumor necrosis factor α (TNF-α) (Angel et al., 2018) (Note: “NF-κβ” in the original text is a notation error, corrected to the universally accepted “NF-κB”).​

In recent years, a growing body of research has linked abnormalities in the actin cytoskeleton to immune system diseases. The actin cytoskeleton regulates inflammatory responses primarily by modulating the activation status of immune cells and the permeability of the endothelial barrier (Lagrue et al., 2013; Lechuga and Ivanov, 2017; Schnoor et al., 2017). Among actin-regulatory molecules, the Arp2/3 complex binds to the side of pre-existing actin filaments to nucleate new branched filaments. To date, its role in inflammatory responses has been thoroughly investigated, with its functional mechanisms in inflammatory cells (e.g., macrophages and neutrophils) well characterized (May 2001). Additionally, significant progress has been made in understanding Arp2/3 complex-mediated cellular processes, including cell motility, vesicle trafficking, cytokine secretion, and the formation and stabilization of cell-cell junctions; concurrently, its specific role in inflammatory responses has been extensively explored (Abu et al., 2014).​Based on the aforementioned research background, this review will focus on the role of the actin cytoskeleton in inflammatory diseases, as well as the functions of the Arp2/3 complex and its regulatory mechanisms in inflammatory responses.

## 2 The role of the actin cytoskeleton in inflammatory response

The actin cytoskeleton is involved in a myriad of essential cellular functions and exerts profound influences on multiple aspects of skin biology. Recent studies have demonstrated that it modulates inflammatory responses by regulating immune cell activation, neutrophil recruitment, and endothelial barrier permeability (Lagrue et al., 2013; Lechuga and Ivanov, 2017). These functions rely on its dynamic properties. The dynamic assembly of actin constitutes the core process underlying cytoskeletal function, encompassing a series of tightly regulated steps (from monomeric to filamentous forms) including nucleation, elongation, branching, and severing.

### 2.1 Nucleation process from monomeric (G-actin) to filamentous (F-actin)

Actin exists in two fundamental forms: globular actin (G-actin) and filamentous multimers (F-actin). G-actin is a spherical protein approximately 5.5 nm in diameter that can bind ATP or ADP, with ATP-bound G-actin (G-actin-ATP) representing the active form for polymerization. Nucleation is the initial and rate-limiting step in the assembly of G-actin into F-actin. Spontaneous nucleation is highly inefficient and thus relies on nucleation factors for mediation. The main nucleation mechanisms include: Arp2/3 complex-mediated nucleation and formin family proteins mediated nucleation.

Among these, the Arp2/3 complex holds a central role—particularly in the context of inflammatory responses. While formin family proteins primarily generate linear actin filaments, the Arp2/3 complex is uniquely tasked with producing branched actin networks: these networks are defining hallmarks of dynamic cell surface protrusions, such as lamellipodia. This specialized function positions the Arp2/3 complex as the primary executor of rapid, directional cytoskeletal remodeling—an essential process supporting key immune cell activities. These activities include migration toward inflammatory cues, formation of immunological synapses, and efficient pathogen internalization. Consequently, the Arp2/3 complex acts as a critical molecular hub: it translates extracellular inflammatory signals into intracellular actin cytoskeletal rearrangements, which in turn drive the functional responses of immune cells.

As a core component of the natural killer (NK) cell cytoskeleton, actin undergoes dynamic interconversion between monomeric globular (G-actin) and polymeric filamentous (F-actin) states via regulated polymerization depolymerization cycles (Mace and Orange, 2014). Upon engagement of surface receptors with cognate ligands, intracellular signaling cascades orchestrate F-actin remodeling, thereby modulating cellular morphology and effector functions. This cytoskeletal reorganization is governed by nucleating factors (e.g., Arp2/3 complex, formins), which directly catalyze actin nucleation to initiate nascent filament assembly and drive cytoskeletal plasticity (Ben-Shmuel et al., 2021).

#### 2.1.1 Arp2/3 complex-mediated nucleation

The Arp2/3 complex, a 225-kDa heptameric protein assembly, is the earliest identified molecule capable of promoting actin filament branching—a function first characterized by Mullins et al. through electron microscopy observations demonstrating its ability to nucleate daughter filaments at a 70° angle from pre-existing actin filaments (Mullins et al., 1998). Of the seven subunits in the Arp2/3 complex, ARP2 and ARP3, exhibit high sequence and structural similarity to actin. In the inactive state, ARP2 and ARP3 adopt a loose ‘long-pitch’ (non-parallel) arrangement. Binding to NPFs (e.g., WASP) induces a conformational shift to a ‘short-pitch’ (parallel) conformation, which mimics the structure of two consecutive actin subunits in F-actin—this provides a template for G-actin polymerization (Muller et al., 2005; Rodnick-Smith et al., 2016). The Arp2/3 complex relies on binding to regulatory proteins known as nucleation-promoting factors (NPFs) to trigger its actin filament nucleation activity. Upon binding to NPF, the conformation of the Arp2/3 complex is changed, and ARP2 and ARP3 mimic the “actin dimer” structure, which provides the polymerization starting point for G-actin to form the “G-actin-Arp2/3″ternary core (Pollard et al., 2000).

#### 2.1.2 Formin family proteins mediated nucleation

Formins are a family of cytoplasmic actin-nucleating proteins that directly bind G-actin-ATP via their C-terminal FH2 (formin homology 2) domain to form a ‘formin-actin dimer’ core, initiating the assembly of unbranched linear F-actin (Evangelista et al., 2003; Kühn and Geyer, 2014). mDia1 and DAAM1, two major actin-regulatory proteins of the formin family, both bind to RhoA and initiate actin polymerization in platelet lysates. In activated platelets, mDia1 translocates to the cytoskeleton in a PI3-kinase-dependent manner. Upon TRAP activation (Gao et al., 2009), DAAM1 can bind to Dvl—a Wnt signaling effector—and thus may play a key role in recruiting and activating RhoA via RhoGef (Steele et al., 2012).

The activation of formins is not dependent on a single GTPase. They can be activated by members of the Rho family, Cdc42, or the Rac family. Rho family (exemplified by RhoA): Driving stress fiber formation in the context of basic morphological maintenance. Rho-mediated activation of formins primarily occurs in the context of cell adhesion and basic morphological maintenance, with a central role in driving stress fiber formation. For instance, RhoA binds to the GTPase-binding domain (GBD) of formins in the Dia family (e.g., mDia1), thereby relieving the autoinhibition mediated by its N-terminal FH3 domain and C-terminal DAD (Diaphanous Autoregulatory Domain). Activated mDia1 polymerizes linear actin filaments through its FH2 domain, which then assemble into stress fibers—structures that contribute to cell adhesion and morphological stability (Tojkander et al., 2012).

Cdc42: Supporting filopodia or lamellipodia formation in the context of cell motility (activation prior to Arp2/3 association). Cdc42 can activate formins such as FMNL1 and FMNL2, promoting their FH1 domain to recruit the profilin-actin complex, accelerating the elongation of linear actin filaments, and supporting the formation of filopodia or lamellipodia. Notably, the effector-binding site of Cdc42 can be competitively bound by formins (e.g., FMNL1) and Arp2/3 activators (e.g., WASP): binding of FMNL1 to Cdc42 displaces WASP and reduces Arp2/3 activation; conversely, WASP binding to Cdc42 also inhibits formin activation, forming a “binary regulatory mode” (Chua et al., 2024).

Rac family (exemplified by Rac1): Constructing branched actin networks in the context of viral infection. Rac1-mediated activation of formins is specific to contexts of viral infection (e.g., vaccinia virus infection). Rac1 is recruited to and activated around virions, which in turn binds to the GBD domain of the formin FHOD1, releasing FHOD1 from its autoinhibited state. FHOD1 exerts critical nucleation and elongation activities through its FH2 domain: it first nucleates the assembly of initial actin filaments; subsequently, Arp2/3 complexes are recruited to these FHOD1-generated “mother filaments” to nucleate the formation of a branched actin network. Concurrently, FHOD1 binds to the “barbed end” of actin filaments to further accelerate their elongation (Alvarez and Agaisse, 2015).

Formin-like gene 1 (FMNL1) regulates actin dynamics via its formin homology domain (FH2). As a key regulatory factor, FMNL1 mediates the migration of T cells to inflamed tissues. Studies have demonstrated that FMNL1-deficient T cells exhibit impaired migration to inflamed tissues (Sigler et al., 2024). In T lymphocytes, agonists of protein kinase Cδ (PKCδ) –such as phorbol myristate acetate (PMA) – potently phosphorylate FMNL1. This phosphorylation event, in turn, regulates cortical actin reorganization at the immune synapse (IS), thereby controlling the polarization of the microtubule-organizing center (MTOC) and multivesicular bodies (MVB). Ultimately, this process enables exosome secretion by helper T cells at the immune synapse, as well as their activation-induced cell death (AICD). (Ruiz-Navarro et al., 2024).

### 2.2 The elongation process of F-actin

Following nucleation, F-actin elongates through the addition of ATP-bound actin monomers (G-actin-ATP) at both ends, with its core characteristic being polarized growth (Carman et al., 2023). F-actin adopts a right-handed helical structure, approximately 7 nm in diameter, and exhibits striking structural and functional disparities between its two ends: Barbed end (plus end): Exposes binding sites for G-actin-ATP, displaying rapid elongation (approximately 10–100 monomers per second). Pointed end (minus end): Primarily binds ADP-bound actin (ADP-actin) and elongates at a slower rate (approximately 1–5 monomers per second) (Pollard and Borisy, 2003).

The primary driving force for elongation stems from the kinetic activity of ATP hydrolysis. Upon binding of G-actin-ATP to the plus end, ATP hydrolysis occurs within approximately 1–5 s, generating ADP and inorganic phosphate (Pi) to form the “F-actin-ADP-Pi” intermediate. Subsequent release of Pi leads to the final stable state of “F-actin-ADP” (Engel et al., 1977).

#### 2.2.1 Accelerating role of profilin

Profilin binds to G-actin-ADP, facilitating the release of ADP and the rebinding of ATP to generate G-actin-ATP. This process indirectly enhances the elongation efficiency of the plus end. Profilins deliver actin monomers to formins and proteins of the VASP family. Most profilins also accelerate nucleotide exchange in actin monomers and counteract the inhibitory effect of ADF/cofilin on nucleotide dissociation from G-actin (Blanchoin and Pollard, 1998). Profilin function is regulated by phosphatidylinositols (e.g., PIP_2_), which act by preventing the formation of actin-profilin complexes (Goldschmidt-Clermont et al., 1991).

In the context of inflammatory regulation, PFN1 can block NF-κB activation by inhibiting the phosphorylation of IκB kinase, thereby suppressing the expression of inflammatory genes. Additionally, PFN1 inhibits IL-17A-induced p65 phosphorylation, whereas intracellular depletion of PFN1 promotes p65 phosphorylation. Taken together, dysregulated PFN1 expression leads to epidermal hyperproliferation and triggers IκBζ-mediated inflammation (Mok et al., 2022). Moreover, profilins can activate Toll-like receptors (TLRs) on the surface of dendritic cells (DCs) to initiate host immune responses and induce the production of pro-inflammatory cytokines (Mansilla et al., 2016).

#### 2.2.2 Sustained regulatory role of formin proteins

During elongation, formin proteins remain continuously associated with the plus end. Through conformational oscillations of their “FH2 domain dimers”, they sustain the recruitment of G-actin-ATP, thereby maintaining efficient linear elongation of F-actin.

Formins restructure the actin cytoskeleton by nucleating new actin filaments and, with the assistance of Profilin-bound G-actin, promoting the elongation of these filaments (Kühn and Geyer, 2014).

In CTLs, cytotoxic granules are transported along microtubules to the IS, where formin (e.g., FMNL1)-regulated actin filaments form a “fusion zone” that restricts granule exocytosis to the synaptic center, ensuring precise perforin/granzyme delivery and avoiding bystander damage (Ritter et al., 2015). In helper T cells, actin remodeling similarly controls polarized secretion of cytokines (e.g., IL-2, IFN-γ) for B cell/macrophage activation. However, FMNL1—another highly expressed formin family member in T cells—is not involved in regulating T cell thymic development or egress. FMNL1 exhibits functional specificity: it primarily mediates the trafficking of activated T cells and acts exclusively in non-lymphoid tissues. T cell migration is impaired only upon FMNL1 depletion (Thompson et al., 2020). During cell migration, FMNL1 also facilitates nuclear translocation through two mechanisms: first, by providing actin filaments for myosin IIA (MyoIIA)-mediated contraction, and second, by generating independent driving forces via actin polymerization at the cell rear (Thompson et al., 2022). It has also been shown that deletion of the Formins family member mDia1 in T cells impairs their migration to lymphoid organs and inflamed skin; In addition, data from B-cell leukemia studies suggest that deficiency of mDia1 also leads to defects in the transepithelial migration (TEM) process (Thompson et al., 2018).

### 2.3 Branch formation of F-actin

The branched structure of the actin filament (F-actin) network is critical for forming specialized cellular structures such as lamellipodia at the cell leading edge and the regions surrounding endocytic structures. Notably, the structural transition and functional specialization of F-actin cannot be achieved solely through its intrinsic polymerization or depolymerization. Instead, this process relies on the Arp2/3 complex, which acts as a key regulatory hub that bridges the fundamental structural properties of F-actin with its context-specific functions. Specifically, the linear filaments of F-actin provide the “pre-existing structural substrate” required for Arp2/3 activity, while the Arp2/3 complex endows F-actin with the capacity to form branched networks—this interdependence underscores that the Arp2/3 complex is not merely an “external regulator” of the actin cytoskeleton, but rather an integral component that enables F-actin to exert specialized roles during inflammatory processes.

This branching is primarily mediated by the Arp2/3 complex, with the process unfolding as follows: The formation of a branched actin network begins with the activation of the Arp2/3 complex. Upstream signals are transmitted by the signaling molecules Rac and Cdc42, which in turn activate downstream proteins—specifically, Wiskott-Aldrich syndrome protein (WASP) and members of the WAVE/SCAR family. Upon binding to the Arp2/3 complex, these proteins form an active “activation complex” (Thompson et al., 2022). This activated complex then associates with existing actin filaments and recruits profilin-bound actin monomers (G-actin) to initiate the synthesis of new actin filaments. The newly formed filaments emerge at a 70° angle relative to the original filaments, gradually assembling into a branched actin network (Blanchoin et al., 2014).

B-cell antigen receptor (BCR) signaling triggers actin cytoskeleton remodeling by stimulating actin severing, polymerization and the nucleation of branched actin networks via the Arp2/3 complex. WASP-family verprolin-homologous protein-2 (WAVE2) enhances microcluster-based BCR signaling and signal amplification, as well as B cell activation in response to antigen-containing cells (Bedi et al., 2023). Upon BCR binds to antigen, the actin cytoskeleton first undergoes transient depolymerization accompanied by increased BCR diffusion. The disassembly of the actin cytoskeleton at the interface between F-actin and the plasma membrane is mediated by cofilin. This disassembly releases BCRs from both antigen unbinding and steric hindrance; this liberated state enables BCR nanoclusters to collide and interact with each other to form micropopulations, a process that helps expand signal transduction (Li et al., 2019).

In migrating T cells, dynamic rearrangements of the branched actin network represent a core driving force behind directional movement. This network expands rapidly at the cell’s leading edge (i.e., the front of the migration axis) through continuous actin polymerization, forming a dense meshwork whose mechanical properties directly propel the plasma membrane outward to generate pseudopodia—structures endowed with both exploratory and anchoring capabilities (de Boer et al., 2023). These pseudopodia not only serve as interfaces for T cell interactions with the surrounding microenvironment (e.g., vascular endothelium, inflamed tissue matrices) but also integrate extracellular chemokine signals (such as CXCL12 and CCL21) to refine migration trajectories, ensuring precise navigation to immune response sites (Vicente-Manzanares et al., 2009).

The Arp2/3 complex and branched actin networks are also implicated in filopodia initiation (Ideses et al., 2008). A current hypothesis posits that Arp2/3-mediated branched structures provide a structural fulcrum for the initial budding of filopodia. This is followed by the sustained activity of Formin family proteins (e.g., FMNL1), which reorganize actin filaments within the branched network into linear arrays, ultimately yielding stable filopodia (Pollard, 2007). Additionally, the synergistic interplay between WASP and the Arp2/3 complex constitutes a critical molecular foundation for immune synapse formation and maintenance. Upon T cell contact with antigen-presenting cells (APCs), T cell receptors (TCRs) recognize peptide-MHC complexes, triggering downstream signaling pathways that induce conformational changes in WASP. Activated WASP then recruits and activates the Arp2/3 complex, which catalyzes the rapid assembly of branched actin networks. This process drives the clustering of T cell surface receptors (e.g., TCR, CD28) at the contact interface, forming the immune synapse core (Rohatgi et al., 1999). Concurrently, dynamic reorganization of the branched network maintains tension homeostasis at the synaptic interface, ensuring sustained TCR signal transduction and directional secretion of cytokines like IL-2. Functional defects in WASP or Arp2/3 impair immune synapse integrity and signaling efficiency, leading to defective T cell activation and compromised initiation of adaptive immune responses (Carlier and Pantaloni, 2007).

### 2.4 The cutting process of F-actin

Cofilin-mediated F-actin severing is the core mechanism for maintaining actin dynamics, effectively preventing excessive F-actin accumulation (McCall et al., 2019). Its mode of action follows a precise “binding-severing-recycling” cycle: First, cofilin specifically binds to ADP-actin subunits within F-actin via its actin-binding domain. Upon binding, conformational changes induce localized mechanical stress concentration. When this stress reaches a threshold, F-actin severs at the binding site, generating two short fragments. These fragments can directly serve as “seeds” to rapidly recruit G-actin-ATP for elongation, accelerating the assembly of new actin filaments with the aid of Formin or Arp2/3 complexes (Jaswandkar et al., 2022).

This cycle endows cofilin with dual functions: it not only eliminates redundant structures by severing old filaments but also provides raw materials for new filament assembly through fragment recycling. Consequently, it efficiently regulates the dynamic balance and functional transitions of the actin network during physiological processes such as leading edge protrusion, trailing edge retraction, and intracellular cargo transport when T cells traverse tissue gaps (Samstag et al., 2013). T cells recognize antigens bound to major histocompatibility complex (MHC) molecules on the surface of antigen-presenting cells (APCs) via their antigen-specific TCR/CD3 complexes. For T cell activation in this process, dual signals are required to drive cofilin phosphorylation: one is the activation signal triggered by TCR/CD3, and the other is the costimulatory signal transmitted through coreceptors such as CD2 and CD28 (Schwartz, 1990; Blanchoin et al., 2000a).

Meanwhile, cofilin-dependent dynamic actin remodeling is a key mechanism underlying immune synapse maturation. Following antigen recognition, cofilin accumulates in a concentric pattern around supramolecular activation clusters (SMACs) at the T cell-APC contact interface. This distribution directly modulates intercellular signal communication and transmission. Experimental evidence demonstrates that inhibiting cofilin function with cofilin-derived cell-permeable peptides—which disrupt cofilin-actin binding—significantly reduces the accumulation of the costimulatory receptor CD2 in the peripheral SMAC (pSMAC) region of the immune synapse, further confirming cofilin’s regulatory role in synaptic structure and function (Eibert et al., 2004).

## 3 Structure and function of the Arp2/3 complex

As a key actin nucleation factor, the Arp2/3 complex plays a central role in maintaining cytoskeletal homeostasis by precisely regulating actin polymerization and the formation of branched actin networks. The branched actin filaments it mediates provide essential mechanical force for a range of cellular physiological processes, including pathogen invasion (Welch et al., 1998), cell differentiation (Bolger-Munro et al., 2019), spindle positioning in meiosis (Sun et al., 2011), and DNA repair (Schrank et al., 2018). The execution of these functions relies on the Arp2/3 complex’s intricate activation mechanisms and regulatory networks, which ultimately support the orderly progression of critical biological processes such as cell migration, division, and morphogenesis.

### 3.1 Mechanism of Arp2/3 complex activation

The core functions of the Arp2/3 complex are manifested in two interconnected processes: first, initiating actin polymerization to drive the assembly of actin monomers into stable core structures (nucleation); second, promoting the formation of branched actin filament architectures to construct branched networks (van Eeuwen et al., 2023; Gautreau et al., 2022). Nucleation acts as the “rate-limiting step” in rapid cytoskeletal reorganization—it overcomes the kinetic barrier of spontaneous actin monomer polymerization, enabling cells to respond swiftly to external signals (e.g., growth factors, mechanical stimuli) and execute physiological activities like migration, division, and morphogenesis through dynamic cytoskeletal remodeling (Gautreau et al., 2022). Conversely, the formation of branched networks endows the cytoskeleton with unique mechanical properties: newly generated “daughter filaments” extend from “pre-existing actin filaments” at a 70° angle, forming dense branched structures that provide structural support and motility for cellular processes such as lamellipodial protrusion and membrane remodeling (e.g., endocytosis) (Alekhina et al., 2017).​

The Arp2/3 complex inherently exhibits weak nucleating activity, and its functional activation requires the precise coordination of multiple factors, including nucleation-promoting factors (NPFs), ATP, actin monomers, and pre-existing actin filaments (Zimmet et al., 2020; Higgs and Pollard, 1999). These factors ensure spatiotemporal control over the complex’s conformation and activity. NPFs are key activators of the Arp2/3 complex, interacting with it via conserved domains to modulate its activity. Based on structural and functional differences, NPFs are categorized into two classes: Type Ⅰ NPFs, including WASP, N-WASP, WAVE, WASH, and JMY (junction-mediated regulatory proteins), are characterized by a VCA domain (comprising verprolin homology, cofilin homology, and acidic domains) (Weaver et al., 2001). The VCA domain acts as a “molecular bridge”: the verprolin domain (or WH2 domain in some Type Ⅰ NPFs) binds free G-actin (monomeric actin) and “delivers” it to the complex, while the central (C) and acidic (A) domains cooperatively engage two sites on the Arp2/3 complex—the Arp2-ArpC1 interface and Arp3 subunit—enhancing activity by stabilizing the complex’s conformation (Ti et al., 2011; Boczkowska et al., 2014). Additionally, the verprolin/WH2 domain binds the barbed end (fast-growing end) of actin monomers, further fine-tuning nucleation and elongation rates (Boczkowska et al., 2014). Type Ⅱ NPFs, typified by cortactin, lack a canonical VCA domain but interact with the Arp2/3 complex via an acidic region (Padrick et al., 2011). Unlike the strong activation by Type Ⅰ NPFs, Type Ⅱ NPFs weakly activate the complex; their core function is to stabilize existing actin branch structures, preventing network disintegration and maintaining cytoskeletal stability (Padrick et al., 2011). Beyond NPFs, Arp2/3 activation depends on other cofactors: ATP binding to Arp3 induces a significant conformational change, exposing a hydrophobic pocket on Arp3—a critical site for the C-domain of Type Ⅰ NPFs to bind and mediate activation (Rodnick-Smith et al., 2016). While nucleotide status (ATP/ADP) minimally affects the complex’s overall structure, ATP binding acts as a “switch” for functional activation, directly regulating nucleating activity (Rodnick-Smith et al., 2016). Free actin monomers provide raw material for nucleation, and pre-existing actin filaments serve as “anchors” for branch formation—by binding pre-existing actin filaments, the complex ensures new branches form at correct positions.

In summary, the Arp2/3 complex establishes a comprehensive regulatory network through its “nucleation-branching” core functions, synergistic activation by NPFs and cofactors, and conformational rearrangements. This network solidifies its role as an indispensable key molecule in regulating cytoskeletal homeostasis and cellular life activities.

### 3.2 Role of Arp2/3 in immune cells

Within the body’s immune regulatory network, the Arp2/3 complex functions as a pivotal molecule in cytoskeletal dynamic regulation, exerting an irreplaceable role in modulating the immune responses of T and B cells. Through its involvement in a spectrum of physiological processes—including cytoskeletal remodeling and dynamic membrane structural changes—it profoundly influences the activation, migration, development, and functional execution of immune cells, thereby serving as a critical bridge linking cellular morphological alterations to immune function realization.​

#### 3.2.1 Multidimensional regulation of T-Cell immune responses by Arp2/3​

As the core effector cells of adaptive immunity, T cells rely heavily on dynamic cytoskeletal remodeling for their proper functionality, with the Arp2/3 complex assuming a central regulatory role in this process. In the context of cell spreading and chemotaxis, relevant studies have underscored the key role of Arp2/3. In the Jurkat T-cell model, depletion of Arp2/3 directly results in a marked impairment of cell spreading on anti-CD3 sheet-like structures, a phenomenon that unequivocally confirms its necessity for the normal spreading capacity of T cells (Xie et al., 2021). Concurrently, inhibition of the WASp/Arp2/3-mediated actin polymerization process leads to a significant suppression of T-cell chemotaxis (Dang et al., 2013). The underlying mechanism lies in the fact that the Arp2/3 complex acts as the central regulator of membrane protrusion formation—structures that serve as the architectural foundation for cellular motility and migration. This explains why the functional status of Arp2/3 directly impacts T-cell chemotactic behavior.

Dysfunction of Arp2/3 also induces alterations in T-cell motility patterns and a reduction in migration efficiency. When Arp2/3 function is impaired in cytotoxic T lymphocytes (CTLs), there is a striking shift in cellular movement from the original lamellipodial-based motility to a bleb-driven migration mode (Dayel and Mullins, 2004). This transition stems from the detachment of connections between the plasma membrane and the actomyosin cortex, a structural aberration that directly culminates in decreased migration rates and potentially compromises the efficiency of CTL-mediated target cell recognition and killing.

Furthermore, Arp2/3 is involved in regulating T-cell motility properties to sustain their biological activity. Research has demonstrated that T cells exhibit distinct motility characteristics in response to varying antigen affinities, with these differences being dependent on modulation by the Arp2/3 complex (Martin et al., 2005). Specifically, the Arp2/3 complex enables T cells to decelerate upon recognition of appropriate antigens. This regulatory mechanism is indispensable for effective T-cell interactions with antigen-presenting cells and the initiation of immune responses, representing a critical link in maintaining T-cell biological activity.

Arp2/3 also plays a significant role in the adaptive regulation of effector CD8^+^ T cells. In IL-2-stimulated T cells, the fitness of effector CD8^+^ T cells is governed by two mechanisms: one involving glucose-dependent ATP production to regulate gene transcription, and the other being the Arp2/3 complex-associated actin remodeling mechanism (Kamnev et al., 2024). These two mechanisms act in concert to modulate the morphological and functional states of effector CD8^+^ T cells, ensuring their effective cytolytic function during immune responses. Notably, Arp2/3 exerts a substantial impact on T-cell development. Loss of the Arp2/3 complex in humans results in a reduction in the number of peripheral naive T cells, thereby disrupting the development of normal T cells (Thompson et al., 2022). This is attributable to the Arp2/3 complex’s prominent role in cytoskeletal dynamic regulation; its depletion may impair critical processes such as T-cell migration, activation, and proliferation within the thymus, ultimately leading to abnormal T-cell development and detrimental effects on the body’s immune reserve and immune response potential.

#### 3.2.2 Key regulatory role of Arp2/3 in B-Cell immune Responses​

B cells, as the primary mediators of humoral immunity within the adaptive immune system, sense antigens and initiate immune responses via the B-cell receptor (BCR). In this process, the Arp2/3 complex exerts a pivotal regulatory role in B-cell immune responses through the modulation of actin remodeling.​

B cells utilize BCRs to perceive antigens; upon binding to antigens presented on the surface of antigen-presenting cells (APCs), a cascade of signaling events and actin remodeling is triggered (Tolar, 2017). Within the primary models governing this process, B cells scan the surface of antigen-presenting cells (APCs) via dynamic actin-rich protrusions mediated by the Arp2/3 complex. These protrusions also generate retrograde actin flow, which drives the centripetal movement of B cell receptor-antigen (BCR-Ag) microclusters and facilitates the formation of the central supramolecular activation cluster (cSMAC). Critically, these Arp2/3-dependent processes amplify BCR signaling, transcriptional responses, and B cell proliferation in response to APC-bound antigens (Ags) (Bolger-Munro et al., 2019). It was further observed that the Arp2/3 complex competes with formins for a limited pool of actin monomers. Inhibition of Arp2/3 not only enhances filopodia formation but also impairs the retraction of BCR-Ag microclusters. Additionally, within B cell synapses, Arp2/3 and formins cooperate to form a loosely connected network that supports efficient antigen extraction: Arp2/3 generates actin foci, which may either promote membrane protrusion or act in synergy with signaling proteins to facilitate antigen extraction; formins produce actin filaments that stabilize these foci; and myosin IIa also contributes to the mechanism of antigen extraction (Roper et al., 2019).

Furthermore, Arp2/3 impacts the strength and precision of BCR signaling by regulating actin’s dynamic reorganization. Studies have revealed that WASP family proteins can activate the Arp2/3 complex, which in turn modulates actin’s dynamic restructuring (Rey-Suarez et al., 2020). This regulatory effect directly influences the diffusion behavior and nanoscale spatial organization of BCRs and their coreceptors during activation. The spatial distribution and interactions between BCRs and their coreceptors are critical determinants of BCR signaling strength and precision, underscoring the key regulatory role of Arp2/3-modulated actin cytoskeletal dynamics in BCR signaling.

In conclusion, the Arp2/3 complex profoundly influences the body’s immune responses by regulating multiple critical physiological processes in both T and B cells. In-depth investigation into its mechanisms of action not only enhances our understanding of immune system regulatory networks but also provides potential therapeutic targets and theoretical foundations for treating related immune disorders.

### 3.3 The functional differences of each subunit of the Arp2/3 complex in immune responses among different types of immune cells

As the core molecular machinery mediating branched actin nucleation, the subunits of the Arp2/3 complex regulate actin cytoskeleton dynamics through division of labor and coordination, collectively supporting the orderly execution of cellular physiological functions. As a key actin-associated protein in the complex, ACTR2 participates in the formation of the actin “protein cap” during the nuclear cycle, providing crucial support for maintaining nuclear structural stability (Wang et al., 2021). ACTR3 and ARPC3, both core components of the complex, take cytoskeleton dynamic regulation as their central function: ACTR3 contributes to essential physiological processes (e.g., cell morphology maintenance, division, and motility) via dynamic modulation (Hu et al., 2021), while ARPC3 further mediates fundamental cellular activities such as cell morphological stabilization and cargo transport—serving as a vital underpinning for cytoskeleton function (Herdoiza Padilla et al., 2019). ARPC1 and ARPC2 play pivotal roles in actin cytoskeleton assembly and nucleation. Beyond mediating actin cytoskeleton assembly and remodeling, ARPC1 acts as a “bridge” to facilitate the interaction between the ARP2/3 complex and WASP family members, laying the groundwork for complex activation and actin branch growth (Hossain et al., 2012). By regulating actin filament nucleation efficiency, ARPC2 directly engages in the core process of cytoskeleton dynamic regulation and serves as a key regulatory factor in branched actin network formation (Frank et al., 2006). ARPC4 and ARPC5 focus on actin cytoskeleton assembly and organization: ARPC4 promotes the assembly of actin monomers into microfilaments, thereby playing a critical role in maintaining cell morphology and constructing cytoskeletal structures (Laboy Cintron et al., 2021). ARPC5 is deeply involved in the organizational process of the actin cytoskeleton, providing support for skeleton dynamics-dependent physiological activities, including cell migration, invasion, and differentiation (Casanova et al., 2014).

The Arp2/3 complex serves as the core molecular machinery governing actin cytoskeleton dynamics. By precisely regulating branched actin nucleation, its subunits play irreplaceable roles in inflammatory microenvironment modulation, epithelial immune barrier maintenance, immune signal cross-pathway integration, and tumor immune infiltration. Dysfunction of these subunits is closely associated with inflammatory diseases, immunodeficiencies, and cancer progression.

In the modulation of the inflammatory microenvironment, multiple subunits contribute to pathological processes through distinct mechanisms: Loss-of-function mutations in the ARPC5 subunit trigger postnatal inflammation and immunodeficiency in mice, ultimately leading to early lethality (Sindram et al., 2023). Concurrently, ARPC5 drives actin polymerization in both the nucleus and cytoplasm of activated CD4^+^ T cells, enhancing T cell receptor (TCR) signaling and the expression of cytokines such as IL-2 and IFN-γ (Ming et al., 2023; Sadhu et al., 2023). Deficiency in ARPC5 results in early-onset systemic inflammation and immune dysregulation, characterized clinically by recurrent respiratory/gastrointestinal infections and enteropathy (Sindram et al., 2023). In the context of diabetic nephropathy (DN), high glucose (HG) induces the upregulation of the ACTR2 subunit in human renal mesangial cells (HRMCs). This upregulation promotes HRMC proliferation, the secretion of proinflammatory cytokines (e.g., IL-6 and TNF-α), and oxidative stress. Notably, inhibiting ACTR2 significantly mitigates these pathological responses by restoring phospholipase D1 (PLD1)-mediated autophagic homeostasis (Zhang et al., 2024; Yun et al., 2021). The ARPC2 subunit enhances the stability of branched actin networks by regulating the binding efficiency between actin filaments and the nucleation core (Frank et al., 2006). Its function is tightly linked to immune cell migration: ARPC2 participates in inflammatory immune dysregulation during myocardial fibrosis, and in vascular smooth muscle cells, it can be activated by H_2_O_2_ via the p38 MAPK pathway to promote cell migration and facilitate inflammatory repair (Al et al., 2013).

In the maintenance of the intestinal epithelial immune barrier, the ACTR3 subunit exhibits “microenvironment-dependent” functionality: In lipopolysaccharide (LPS)-induced acute intestinal epithelial injury, Dragon’s Blood (DB) activates ACTR3 through the “FAK-DOCK180-RAC1-WAVE2-ACTR3” pathway. This activation drives actin cytoskeleton assembly, upregulates the expression of tight junction proteins (e.g., occludin), and triggers NF-κB-mediated anti-inflammatory responses—ultimately reversing intestinal epithelial barrier (IEB) damage and reflecting ACTR3’s protective role (Liu et al., 2024). In contrast, in the chronic inflammatory disorder ulcerative colitis (UC), clinical studies demonstrate a positive correlation between ACTR3 expression in intestinal mucosa and disease severity. *In vitro* experiments using a TNF-α-induced inflammatory model of colonic epithelial cells (NCM-460) further confirmed that knockdown of ACTR3 reduces the expression of apoptosis-related markers (e.g., Caspase-3 and Bax). These findings suggest that ACTR3 exacerbates the pathological progression of UC by promoting intestinal epithelial cell (IEC) apoptosis (Zhang et al., 2019). This functional dichotomy clearly reflects the regulatory priority of microenvironmental signals on subunit activity.

In the cross-pathway integration of immune signals, individual subunits ensure the order and efficiency of signal transmission through diverse mechanisms: ACTR3 acts as a “signaling hub”: On one hand, it is activated by IL-6 and IL-8 secreted by cancer cells, which engage the histone methyltransferase MLL1/Menin-ACTR3 or IL-6/P-STAT3-ACTR3 pathways (Figure 2) to regulate actin dynamics and influence T cell migration and infiltration (Nair et al., 2024). On the other hand, ACTR3 binds to ERK3 and undergoes Ser418 phosphorylation, which activates Cdc42 to further enhance Arp2/3 complex activity—forming a regulatory axis of “immune cytokine-kinase signal-actin remodeling” (Bogucka-Janczi et al., 2023). ACTR2 indirectly participates in immune signal regulation by maintaining nuclear structural stability: Yes-associated protein 1 (YAP1) and transcriptional coactivator with PDZ-binding motif (TAZ1) directly transcriptionally regulate ACTR2 to preserve nuclear envelope integrity (Sladitschek-Martens et al., 2022). Impairment of the nuclear envelope leads to aberrant activation of nuclear inflammatory signals (e.g., NF-κB), indicating that ACTR2 serves as a “structural scaffold” for the orderly transmission of immune signals (Wang et al., 2021). The ARPC1B subunit focuses on adaptive immune regulation: It binds to Wiskott-Aldrich syndrome protein (WASP) to modulate B cell actin polymerization, reducing “tonic signaling” in B cells to prevent autoimmune responses (Leung et al., 2021). Additionally, ARPC1B is essential for cytotoxic T lymphocytes (CTLs) to maintain their cytolytic activity (Randzavola et al., 2019).

Aberrant expression of Arp2/3 complex subunits is a central mediator of cancer progression in the context of tumor immune infiltration and immune escape: In hepatocellular carcinoma (HCC), family members including ACTR2, ACTR3, ARPC1A, ARPC1B, ARPC2, ARPC3, ARPC4, ARPC5, and ARPC5L are upregulated (Huang et al., 2022; Huang et al., 2021). Higher expression of these subunits correlates significantly with poor patient survival and advanced cancer stages, with ACTR3, ARPC2, and ARPC5 showing positive correlations with immune cell infiltration. In colorectal cancer, ARPC4 enhances cancer cell invasiveness by promoting actin monomer assembly; its overexpression also inhibits T cell infiltration and facilitates immune escape via modulation of the p53 signaling pathway (Xu et al., 2020; Otsubo et al., 2004). In prostate cancer, ARPC1A is transcriptionally regulated by STAT3: It not only supplies energy to cancer cells by enhancing oxidative phosphorylation but also maintains an immunosuppressive microenvironment by inhibiting ferroptosis—creating favorable conditions for cancer progression (Zhou et al., 2025; Ji et al., 2022). ARPC5 is considered to be an oncogene in the occurrence and development of a variety of tumors, and its upregulation is significantly associated with poor prognosis of multiple myeloma and infiltration of a variety of immune cells (Huang et al., 2021; Xiong and Luo, 2018; Chang et al., 2012). It can promote the growth, invasion, metastasis and angiogenesis of tumor cells through CPEB2 regulating its mRNA stability (Zeng et al., 2023), TAGLN2 affecting the MEK/ERK pathway (Gao et al., 2024), KLF4-mediated upregulation of ADAM17 expression (Qu et al., 2023), and YAP regulation (Lui et al., 2022). It is a potential target for tumor treatment. It also provides directions for the research of immune diseases and cancer treatment strategies.

## 4 Inhibition and activation of the Arp2/3 complex

In the intricate regulatory network governing cellular life processes, the Arp2/3 complex serves as a pivotal nucleating factor that mediates the formation of actin branched networks. Notably, the activation and inhibition of the Arp2/3 complex are equally pivotal at the functional level, as neither process can be overlooked in maintaining cytoskeletal homeostasis. Excessive activation of the Arp2/3 complex triggers aberrant proliferation of actin branches, disrupting the establishment of normal cellular morphology and impeding the transduction of essential signaling pathways. Conversely, insufficient inhibition of the complex impairs the cell’s adaptive responses to microenvironmental cues, ultimately leading to cellular dysfunction. Thus, dissecting the inhibitory mechanisms of the Arp2/3 complex holds scientific significance comparable to investigating its activation mechanisms.

### 4.1 Function of Arp2/3 inhibitors in immune cells

**FIGURE 1:**
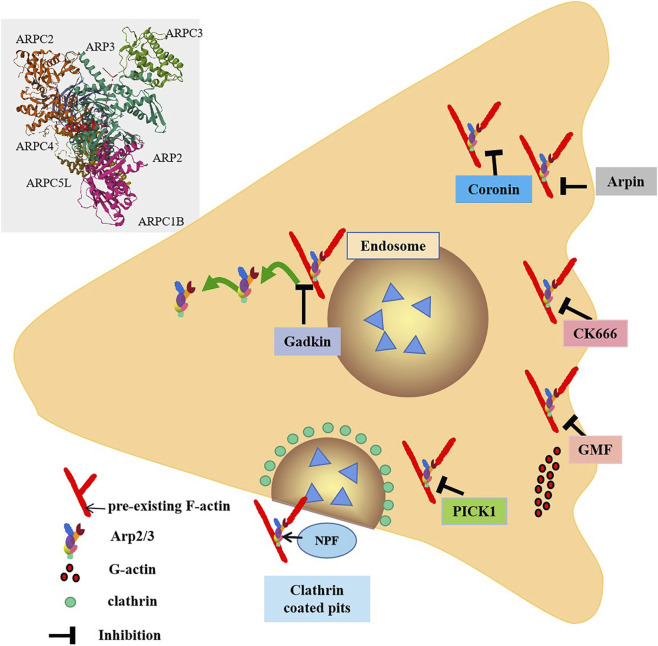
Inhibitors of the Arp2/3 complex. The Arp2/3 complex is composed of seven subunits. NPF binds to the Arp2/3 complex and activates it. From the Arp2/3 complex extend branched actin fibers that drive membrane invagination. Arpin inhibits the Arp2/3 complex by directly competing with WAVE for binding sites. Gadkin interferes with the interaction between nuclear activators and the Arp2/3 complex in a competitive manner. Coronin directly binds to ArpC2, the subunit of Arp2/3 complex, in monomeric form through its C-terminal sequence, thereby inhibiting the activity of Arp2/3 complex. GMF exerts branching effects by interfering with the branch connections between the Arp2/3 complex and actin filaments. PICK1 inhibits the process of F-actin nucleotide generation mediated by the Arp2/3 complex, thereby affecting the assembly and growth of actin filaments and further influencing endocytosis and vesicle transport. CK666 can specifically bind to the subunits Arp2 and Arp3 of the Arp2/3 complex, thereby inhibiting the conformational changes of the Arp2/3 complex and blocking its function.

In cellular processes, inhibition of the Arp2/3 complex is equally critical its activation. Known inhibitors of the Arp2/3 complex include coronin (Sokolova et al., 2007), Arpin (Dang et al., 2013), GMF (Boczkowska et al., 2013), gadkin (Maritzen et al., 2012), C-kinase interacting protein (PICK1) (Rocca et al., 2008) and CK666 (Hetrick et al., 2013) (Figure 1).

#### 4.1.1 Coronin

Coronin stands as a widely studied inhibitor of the Arp2/3 complex. In its monomeric form, coronin directly binds to ArpC2, a subunit of Arp2/3 complex, via its C-terminal sequence, thereby suppressing the activity of Arp2/3 complex (Sokolova et al., 2007). The coronin protein Crn1 in yeast exerts dual regulatory effects on the Arp2/3 complex. On one hand, it can inhibit Arp2/3 activity; On the other hand, at low concentrations, Crn1 is capable of activating Arp2/3 through its central acidic (CA) domain. This CA domain is located to a specific region and exhibits high similarity to the acidic domain of the Wiskott-Aldrich syndrome protein (WASP). Such similarity may enable the CA domain of Crn1 to form an amphipathic helix, which interacts with Arp2/3 complex to induce a conformational change that promotes its activation (Liu et al., 2011; Humphries et al., 2002). In immune cells, the functions of neutrophils are under highly complex regulatory mechanisms, where both coronin and the Arp2/3 complex serve as key regulators. Coronin 1A, in particular, is ubiquitously expressed in neutrophils and participates in various aspects of neutrophil physiology. It engages in specific interactions with the cytoplasmic tail of CD18, an essential subunit of β2 integrins, thereby modulating crucial processes such as neutrophil adhesion, spreading, and migration during innate immune responses. A compelling example comes from a *Helicobacter pylori* mouse infection model: in Coronin 1A-deficient (Coro1A^−/−^) mice, neutrophil infiltration into the gastric mucosa was significantly diminished, which subsequently resulted in alleviated gastric inflammation (Pick et al., 2017). This finding underscores the critical role of Coronin 1A in orchestrating neutrophil trafficking.

#### 4.1.2 Arpin

Arpin is a recently identified inhibitor of the Arp2/3 complex (Figure 1). Functioning under the regulation of Rac1, Arpin is thought to act by directly competing with WAVE for binding sites on the Arp2/3 complex. In T lymphocytes, Arpin functions as a critical regulator by effectively terminating the Rac1-WAVE-Arp2/3 positive feedback loop upon its presence. Through direct binding to the Arp2/3 complex, Arpin inhibits its nucleation activity, thereby restricting excessive lamellipodia formation—a process essential for fine-tuning T cell migration. During migration, T lymphocytes must strike a precise balance between speed and directionality; unchecked lamellipodia expansion would lead to uncoordinated protrusive activity, impairing their ability to navigate efficiently through complex tissue microenvironments (Dang et al., 2013). Arpin-mediated inhibition ensures that lamellipodia dynamics are tightly controlled, enabling T cells to maintain directed motility and traverse tissues with enhanced precision.

#### 4.1.3 Gadkin

Gadkin, an inhibitory protein with a specific domain, primarily binds to the Arp2/3 complex via this domain, competitively interfering with the interaction between NPF and the Arp2/3 complex (Chánez-Paredes et al., 2019). Notably, this process does not impair the intrinsic nucleation activity of the Arp2/3 complex, meaning its core function of promoting actin polymerization remains intact. In cellular contexts lacking gadkin, the Arp2/3 complex exhibits increased translocation to the plasma membrane, which may enhance cytoskeletal reorganization and thereby boost cell migration capacity (Maritzen et al., 2012).

As an inhibitor of Arp2/3 complex, Gadkin regulates dendritic cell (dc) function by regulating Arp2/3 activity. It has been shown that Gadkin-deficient DCs show no apparent abnormalities during differentiation and that their mature phenotype, including the expression of costimulatory molecules, remains intact. However, intracellular actin polymerization was clearly enhanced, consistent with a mechanism by which disinhibition of the Arp2/3 complex promotes the assembly of branched actin networks. Further functional analysis revealed that Gadkin deletion resulted in marked impairment of DC migration *in vitro*. Remarkably, the migration of Gadkin-null DCS remained unchanged *in vivo*, suggesting the existence of some compensatory mechanism such as functional redundancy between other Arp2/3 regulators or adaptive regulation mediated by tissue microenvironment signals (Schachtner et al., 2015). Therefore, the important physiological function of dcs migrating to secondary lymphoid organs to activate T cells is preserved. Thus, gadkin plays an crucial role in regulating Arp2/3 complex distribution and cell migration.

#### 4.1.4 GMF

GMF (Gelsolin family protein) contains an actin depolymerase homolog (ADF-H) domain, placing it within the ADF-H family. GMFs inhibit Arp2/3 activation by competing with the VCA domain of NPF WASP for binding to Arp2/3 complex (Gandhi et al., 2010). The ADF-H domain of GMFs features a surface-specific site that binds to the first actin subunit of actin filaments. This domain shares homology with the surface of cofilin that specifically interacts with F-actin. Consequently, GMFs exert a debranching function by disrupting the branched connection between the Arp2/3 complex and actin filaments, mirroring the mechanism of cofilin (Ydenberg et al., 2013). In experimental autoimmune encephalomyelitis (EAE) models, GMF-deficient mice exhibit significantly reduced expression levels of several key cytokines (including pro-inflammatory cytokines) and chemokines within the central nervous system, encompassing both brain and spinal cord tissues. This finding suggests that GMF may regulate the production of these inflammatory mediators and thereby participate in the initiation and progression of autoimmune inflammatory responses in the EAE model (Zaheer et al., 2007).

#### 4.1.5 PICK1

PICK1 (C-kinase interacting protein) competes with the N-WASP-VCA domain for binding site of Arp2/3 complex and inhibits actin polymerization induced by this activator (Rocca et al., 2008). It has been demonstrated that PICK1 interacts with F-actin and the Arp2/3 complexes via its BAR domain. This interaction suppresses Arp2/3-mediated F-actin nucleation, thereby influencing actin filament assembly and elongation. These findings suggest that PICK1 may play a significant role in regulating the dynamic reorganization of the cytoskeleton, including processes such as cell morphological changes, motility or division (Madasu et al., 2015).

#### 4.1.6 CK666

CK666 is a compound that specifically binds to the Arp2/3 complex at the interface between the Arp2 and Arp3 subunits (Figure 1). This binding inhibit the conformational changes required for Arp2/3 activation, thereby blocking its function. Importantly, CK666 did not affect the binding of NPFs to Arp2/3 complex (Hetrick et al., 2013). By inhibiting Arp2/3 activity, CK666 may indirectly enhance the activity of other actin nucleators, such as formins. When Arp2/3 is inhibited, the availability of actin monomers increases, and these monomers are utilized by formins to generate new actin structures (Henson et al., 2015). This suggests that CK666 influences actin network reorganization and dynamics by regulating the balance between Arp2/3 and formins. In immune cells such as neutrophils, co-incubation with CK-666 has been shown to exert significant effects. For instance, in a study investigating neutrophil extracellular trap (NET) formation, CK-666 reduced 12-phorbol 13-myristate acetate-induced NET formation *in vitro* (Ding et al., 2022). It not only abrogated F-actin polymerization but also led to intracellular retention of NETs. In mice subjected to cecal ligation and puncture (CLP), inhibition of Arp2/3 by CK-666 decreased NET formation on circulating neutrophils and within the bronchoalveolar space (Ding et al., 2022). These findings demonstrate that CK-666 can effectively inactivate Arp2/3 in immune-relevant cell types, thereby impacting crucial immune-related processes such as NET release.

### 4.2 The regulatory effect of upstream activators on Arp2/3 in immune cells

#### 4.2.1 Rho-family GTPases

Upstream of actin regulators lies a network of signaling pathways that precisely respond to extracellular cues and coordinate dynamic cytoskeletal rearrangements, with Rho-family GTPases serving as core regulatory molecules that function through dynamic transitions between an active GTP-bound state and an inactive GDP-bound state: when bound to GTP, these molecules are activated to initiate downstream signaling, whereas hydrolysis of GTP to GDP switches them to an inactive state, terminating signaling. This “on/off” mechanism underpins their functionality, enabling precise control over the organization and remodeling of the actin cytoskeleton—processes critical for T-cell activation, polarization, and migration (Heasman and Ridley, 2008); for instance, following antigen recognition by T cells, rapid actin cytoskeletal reorganization is required to form immunological synapses, a process coordinated by the state transitions of Rho-family GTPases.

The activity of Rho-family GTPases is subject to tight dual regulation by two classes of molecules: Guanine nucleotide exchange factors (GEFs) act as “activators,” promoting the replacement of GDP with GTP on Rho-family GTPases, thereby converting them from an inactive GDP-bound state to an active GTP-bound state and initiating downstream cytoskeletal regulatory signals, while GTPase-activating proteins (GAPs) function as “inhibitors,” enhancing the intrinsic GTPase activity of Rho-family GTPases to facilitate the hydrolysis of bound GTP to GDP, returning the molecules to an inactive state and thus terminating signaling (Rossman et al., 2005).

##### 4.2.1.1 Rac1

As a key member of the Rho-family GTPases, Rac1 plays an irreplaceable role in actin polymerization and pseudopod formation in T cells, serving as a fundamental driver of T-cell motility and immunological synapse formation (Tybulewicz and Henderson, 2009).

**FIGURE 2:**
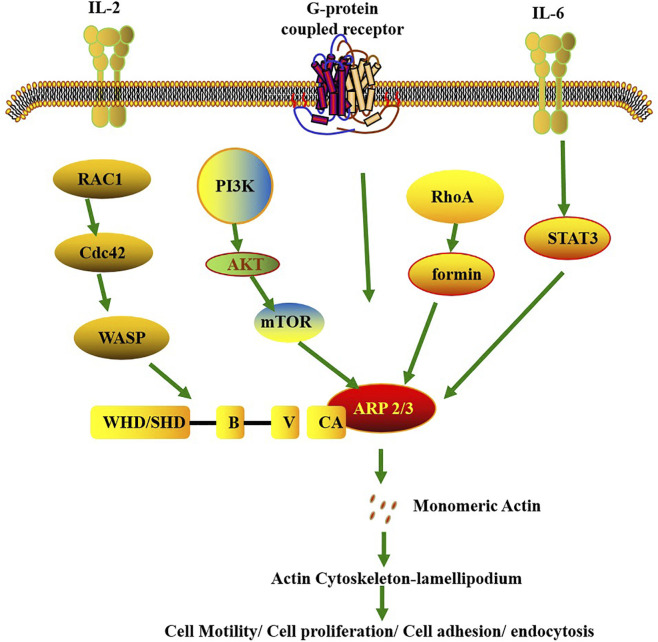
Signaling pathways involved in the regulation of Arp2/3 activity. It leads to the activation of Arp2/3 through the upstream signals of membrane proteins and has extensive physiological and pathological effects. The membrane protein G protein-coupled receptor recruits WRC to the cell membrane, thereby triggering the polymerization of the actin cytoskeleton. The IL-6/8/PSTAT3/ARP3 signaling axis regulates cell migration. The Arp2/3 complex can be activated by external signals, and this activation depends on the participation of the Rac1 protein. The Rac1-WAVE-Arp2/3 complex is locally activated. In addition, mTORC2 forms a signaling axis through Akt and enhances the activity of the Arp2/3 complex. These are crucial for cell migration, expansion, adhesion, division and invasion.

Specifically, upon antigen stimulation of the T-cell receptor (TCR) on the T-cell surface, signals are transduced to intracellular guanine nucleotide exchange factors (GEFs)—among which Vav1 acts as a critical activating molecule. These GEFs (e.g., Vav1) induce a conformational change in Rac1, which is originally in an inactive GDP-bound state, promoting the displacement of GDP by GTP and thereby activating Rac1. Activated Rac1 then recruits WAVE2 (WASP-family verprolin-homologous protein 2), an important activator of the Arp2/3 complex (Figure 2). WAVE2 binds to and activates the Arp2/3 complex, which in turn efficiently induces branched polymerization of actin filaments (Nolz et al., 2006). This branched actin polymerization generates the force required for T-cell spreading and facilitates the formation of membrane protrusions—structures essential for T cells to execute their functions during immune responses (Nolz et al., 2006).

This Rac1-mediated molecular cascade is indispensable for efficient T-cell activation, as experimental evidence demonstrates that Rac1 deficiency leads to significant functional impairments in T cells: TCR clustering at the immunological synapse is severely disrupted, impairing effective antigen signal transduction, and the production of IL-2—a key cytokine for T-cell activation—is markedly reduced, ultimately compromising T-cell proliferation and effector functions (Saoudi et al., 2014; Guo et al., 2008).

Additionally, Rac1 plays a pivotal role in T-cell migration: by regulating integrin-mediated cell adhesion and coordinating dynamic actin cytoskeletal remodeling, it ensures the unimpeded migration of T cells within complex lymphoid organ microenvironments; for example, in lymphoid tissues, T cells must constantly interact with endothelial and stromal cells, and Rac1 enhances cell adhesion by modulating the activation state of integrins (e.g., switching from low to high affinity) while driving actin polymerization to form pseudopods and related structures that provide migratory force, ensuring that T cells can efficiently patrol, localize, and participate in immune responses within lymphoid organs (Fritz and Pertz, 2016).

##### 4.2.1.2 Cdc42

Cdc42 is primarily involved in regulating actin dynamics and cell polarity. It enhances cell migratory capacity and antigen-scanning efficiency by controlling filopodia formation (Dustin and Cooper, 2000), and interacts with the Par3/Par6 polarity complex and atypical protein kinase C (aPKC) to establish anteroposterior polarity during T-cell migration (Etienne-Manneville, 2004). Additionally, Cdc42 plays a pivotal role in centrosome orientation and TCR clustering at immunological synapses, ensuring efficient signal transduction and sustained T-cell activation (Billadeau et al., 2007). Conversely, loss of Cdc42 impairs the ability of T cells to form stable interactions with antigen-presenting cells (APCs), resulting in defective immune responses (Beyersdorf et al., 2015).

##### 4.2.1.3 ROCK

As a key member of the Rho family of GTPases, RhoA exerts its functions primarily through the Rho-associated kinase (ROCK) pathway, with its core role being the precise regulation of actin-myosin contractility—a process critical for T-cell contraction and the maintenance of immunological synapse stability (Tzima et al., 2002).

By activating the ROCK pathway, RhoA induces the formation of intracellular stress fibers and enhances cortical tension. Stress fibers, rigid structures composed of actin and myosin, provide mechanical support to the cell, while heightened cortical tension strengthens the mechanical integrity of the cell membrane. This allows T cells to maintain sufficient structural stability to withstand external pressures when traversing tight vascular endothelial barriers or interstitial spaces, ensuring their unimpeded passage through narrow physiological channels (Lammermann et al., 2008). This process is essential for T cells to migrate from the bloodstream into lymphoid organs or inflammatory sites, where they carry out immune surveillance and effector functions.

Additionally, RhoA modulates the phosphorylation state of myosin light chains via the ROCK pathway. Phosphorylation of myosin light chains serves as a key signal that triggers actin-myosin interactions, generating contractile forces that drive T-cell morphological remodeling—such as retraction of the cell body and extension of the leading edge—facilitating the complex morphological changes required for transendothelial migration (Lammermann et al., 2008; Smith et al., 2003). RhoA also plays an indispensable role in maintaining the stability of immunological synapses. These specialized signaling structures, formed upon contact between T cells and antigen-presenting cells (APCs), directly determine the duration and intensity of T-cell receptor (TCR) signaling. By regulating actin-myosin contractility, RhoA preserves the structural integrity of the immunological synapse, preventing premature dissociation. This ensures the sustained, efficient transmission of TCR signals, enabling T cells to initiate normal effector functions post-activation, such as proliferation, differentiation, and cytokine secretion (Rougerie and Delon, 2012; Ridley, 2011).

In summary, RhoA-mediated regulation of actin-myosin contractility through the ROCK pathway not only provides the mechanical force and structural support necessary for T-cell migration across barriers but also underpins the stability of immunological synapses and the effective transduction of TCR signals.

#### 4.2.2 WASP family protein

WASP proteins are the most well-studied members of NPF family, defined by a C-terminal VCA (Verprolin homology-Central-Acidic) domain that activates the Arp2/3 complex (Marchand et al., 2001; Campellone et al., 2023). As the smallest functional region of the WASP protein, the VCA segment is nonetheless sufficient to activate the Arp2/3 complex, thereby promoting actin polymerization in the cytoskeleton. Upon activated, WASP (Wiskott-Aldrich syndrome protein) triggers the Arp2/3 complex to initiate actin filament nucleation (Burianek and Soderling, 2013). WASP acts as a bridge connecting the Arp2/3 complex to intracellular signaling pathways. The availability of actin monomers helps regulate the branching density generated by the Arp2/3 complex, while the presence of preexisting actin filaments ensures that the Arp2/3 complex, when activated, correctly generates new branching structures (Mullins et al., 2018; Lappalainen et al., 2022). This process is critical for the dynamic reorganization of the cytoskeleton.

Activation of the T-cell receptor (TCR) by peptide-loaded major histocompatibility complex (MHC) molecules triggers a well-orchestrated signaling cascade within the intracellular region of the TCR complex (Babich and Burkhardt, 2013). Initially, the Src family kinase Lck mediates the phosphorylation of immunoreceptor tyrosine-based activation motifs (ITAMs); these phosphorylated ITAMs then serve as docking sites to recruit the tyrosine kinase Zap70. Upon recruitment, Zap70 sequentially phosphorylates two key adaptor proteins—linker for activation of T cells (LAT) and SLP-76—which act in concert to form a functional binding site for phospholipase Cγ1 (PLCγ1) (Carrizosa et al., 2009). Additionally, SLP-76 recruits Vav1 and Itk, two critical regulators of downstream signaling pathways. Specifically, Vav1 activates the small GTPases Rac and Cdc42, which in turn modulate the activity of WAVE (WASP-family verprolin-homologous protein) and WASp (Wiskott-Aldrich syndrome protein), respectively. Once activated, WASp releases its VCA (Verprolin homology, Central, Acidic) domain: this domain binds monomeric actin via its Verprolin homology (V) region and interacts with the Arp2/3 complex through its Central-Acidic (CA) region, collectively driving the nucleation of branched actin filaments (Blanchoin et al., 2000b).

WAVE2 is capable of activating the Arp2/3 complex and, in collaboration with WASp/N-WASp, participates in Arp2/3 complex-dependent actin polymerization—a process that ultimately promotes the generation of actin retrograde flow. Depletion of WAVE2 leads to a reduction in the velocity of actin retrograde flow; concurrently, it suppresses B-cell receptor (BCR) signaling (including the signal amplification cascade) and results in decreased expression of CD69, a key activation marker of primary B cells.​Furthermore, at the immune synapse, WAVE2 depletion significantly reduces the formation of actomyosin arcs. In integrin-mediated cellular processes, WAVE2 also mediates fibronectin (FN)-induced actin cytoskeleton remodeling and B-cell spreading, thereby contributing to the interaction between B cells and the extracellular matrix (ECM) (Bedi et al., 2023).

## 5 Research consensus and controversy on Arp2/3 in immune cells

Across studies, a clear consensus has emerged: the Arp2/3 complex is an indispensable hub for cytoskeletal dynamics that underpins the core functions of T and B cells (the pillars of adaptive immunity), aligning with the overarching framework that “spatiotemporal control of actin branching is a universal driver of immune cell activation and effector function.” In T cells, all key studies confirm Arp2/3-mediated actin branching is required for immunological synapse (IS) maturation (e.g., TCR clustering, MTOC polarization) (Xie et al., 2021; Dang et al., 2013; Nolz et al., 2006), directed migration (via lamellipodia formation) (Dang et al., 2013; Vitriol et al., 2015), and effector function (e.g., CTL cytotoxicity, IL-2 secretion) (Kamnev et al., 2024; Saoudi et al., 2014); for instance, Rac1 knockout (Saoudi et al., 2014) and WASP deficiency (Blanchoin et al., 2000b) studies independently demonstrate that impaired Arp2/3 activation disrupts TCR signal transduction, validating Arp2/3 as a downstream convergence point for T cell activation pathways. In B cells, consistent findings show Arp2/3 drives BCR microcluster centripetal movement (via retrograde actin flow) to form the cSMAC (Bolger-Munro et al., 2019; Goley and Welch, 2006), amplifies BCR signaling (Rey-Suarez et al., 2020), and enables efficient antigen extraction from APCs (Roper et al., 2019), while Arp2/3 inhibitors (e.g., CK666) or WAVE2 depletion (Bedi et al., 2023) uniformly reduce B cell spreading and CD69 expression—confirming its non-redundant role in B cell activation. These similarities reinforce the “cytoskeletal checkpoint” model of adaptive immunity, where Arp2/3 translates extracellular antigen signals into intracellular actin remodeling to dictate the efficiency of immune cell activation.​

Reported discrepancies, however, do not contradict this core consensus but instead reveal the complexity of Arp2/3’s regulatory network, stemming primarily from cell type specificity, microenvironmental context, or subunit subtype heterogeneity. For dendritic cells (DCs), studies on migration show conflicting results: deletion of Gadkin (an Arp2/3 inhibitor) impairs DC migration *in vitro* (due to enhanced Arp2/3-mediated actin polymerization) (Schachtner et al., 2015), yet *in vivo* DC migration to secondary lymphoid organs remains intact—attributed to compensatory mechanisms (e.g., functional redundancy of cortactin, tissue-derived signals) absent in simplified *in vitro* systems, which fits the broader “multi-layered immune cell migration” model where tissue-specific cues override Arp2/3 dependence via alternative cytoskeletal regulators. Meanwhile, conserved Arp2/3 subunits exhibit divergent functions: ARPC5 deficiency causes severe systemic inflammation in humans (Sindram et al., 2023), but its homolog ARPC5L knockout has no obvious immune phenotype (Abella et al., 2016); ARPC1A drives cancer cell proliferation (Zhou et al., 2025), while ARPC1B is critical for B cell tonic signaling and platelet function (Randzavola et al., 2019; Leung et al., 2021). These differences suggest subunit subtypes assemble into cell-specific Arp2/3 variants, challenging the earlier view of a single, universal complex and highlighting the need for subtype-specific cytoskeletal regulation frameworks.​

Beyond immune cell-specific roles, the Arp2/3 complex dominates the nucleation of branched actin filaments (e.g., in lamellipodia and endocytic patches), and understanding of filopodia formation has evolved from the “convergent extension” model to the “tip complex self-assembly” model. Svitkina et al. used live-cell imaging and electron microscopy to show actin filaments elongate via anti-capping proteins (e.g., VASP), aggregate into Λ-shaped precursors, and mature into filopodia with fascin-mediated bundling (Svitkina et al., 2003)—emphasizing pre-existing filament convergence over *de novo* Arp2/3 nucleation. Yang et al. further discovered that the formin family member mDia2 (via N-terminal interaction with the SH3 domain of Abi1) localizes to the cell membrane leading edge, generating actin filaments for lamellipodia (preventing capping to sustain protrusion) while condensing these filaments into bundles within lamellipodia to form filopodia (BiolYang et al., 2007), establishing formins as core links between the two membrane protrusion types. In 2010, Lee et al. reconstituted filopodia-like structures (FLSs) *in vitro* using frog egg extracts and PI(4,5)P2-containing lipid bilayers, defining the protein recruitment sequence (toca-1 → N-WASP → Arp2/3 complex + actin → formins/VASP → fascin) and finding Arp2/3 is essential for FLS initiation (inhibition reduces FLS formation) but not elongation (formins drive monomer addition) (Lee et al., 2010)—integrating Arp2/3-mediated nucleation and formin/VASP-mediated elongation to fill gaps in earlier filopodia biogenesis models.​

As core actin nucleators, Arp2/3 and formins exhibit a “cooperation-competition” dynamic balance shaped by cell state and regulatory factors, with Profilin acting as a key link. In cooperation, Chua et al. (Chua et al., 2024) used actin waves as a model to show the formin isoform FMNL1 is recruited to the membrane 2.3 ± 0.7 s before Arp2/3; FMNL1 polymerizes linear actin filaments via its FH1-FH2 domain, providing “mother filaments” for Arp2/3-mediated branched nucleation, while Profilin delivers profilin-actin complexes to FMNL1’s FH1 domain via its polyproline-binding surface—slightly reducing formin nucleation efficiency but increasing linear filament elongation by up to 10-fold (Henty-Ridilla and Goode, 2015) to supply stable substrates. Cao et al. (Cao et al., 2020) further found that the nucleation-promoting factor SPIN90 recruits the formin mDia1 to the barbed ends of Arp2/3-nucleated filaments and forms a ternary complex (boosting nucleation efficiency 5-fold vs. SPIN90-Arp2/3 alone), creating composite filaments with SPIN90-Arp2/3 at the pointed end and mDia1 mediating elongation at the barbed end; Profilin’s dual binding surfaces (actin-binding to reduce excessive Arp2/3 branching, polyproline-binding to deliver monomers to mDia1) further optimize this cooperation, regulating cortical actin’s mechanical properties and turnover efficiency. In competition, Rotty (Rotty and Bear, 2014) and Suarez (Suarez et al., 2015) proposed the “Profilin-mediated actin monomer competition” model: Profilin competes with nucleation-promoting factors (NPFs, e.g., WASP) for G-actin to inhibit Arp2/3, while preferentially delivering profilin-actin complexes to formins/Ena/VASP—their anti-capping activity confers a competitive advantage to linear filament networks, enabling simultaneous assembly of stress fibers/filopodia and lamellipodia. Different species exhibit compensatory mechanisms: fission yeast rely on formins after Arp2/3 knockout, while mammalian fibroblasts depend on Profilin-1 and Ena/VASP (Rotty et al., 2015). Chua et al. revised this model to the “dynamic reset of active Cdc42”model, noting competition occurs across two timescales—second-scale (Arp2/3 inhibition instantly increases formin recruitment) and long-term (formin activation reduces Arp2/3 recruitment)—independent of the actin monomer pool. This competition is driven by active Cdc42 (reset every 20–30 s) via two mechanisms: mutually exclusive binding of FMNL1 and WASP to Cdc42, and Arp2/3-recruited lipid phosphatase SHIP1 exerting negative feedback on PI(3,4,5)P3 to limit active Cdc42; Profilin maintains formin basal activity to ensure immediate responses to Arp2/3 activity changes (Chua et al., 2024).​

In addition to mediating cooperation and competition, Profilin exerts global regulation via functional specialization (its actin-binding surface inhibits Arp2/3, while its polyproline-binding surface activates formins/Ena/VASP (Guérin et al., 2025)) and multi-factor synergy (e.g., collaborating with thymosin β4 to target G-actin delivery to formins in lamellipodia). Together, these components form a multi-level regulatory network that ensures the actin network meets cellular structural demands for branched and linear filaments while adapting to dynamic intracellular and extracellular signals.

In summary, the Arp2/3 complex is responsible for branched filament nucleation (e.g., in lamellipodia and endocytic patches), while the formin family mediates linear filament nucleation and elongation (e.g., in filopodia and stress fibers); these two nucleator families maintain the balance of the actin network by jointly providing structural foundations and competing for the distribution of regulatory resources.

## 6 Summary and future directions

The Arp2/3 complex is a key effector governing eukaryotic actin cytoskeleton remodeling, with roles extending far beyond cell morphology maintenance to deep integration in the entire immune response cascade:by mediating the precise assembly and dynamic regulation of branched actin filaments, it acts as a core structural and functional hub enabling immune cells to sense extracellular signals and execute biological functions, and in adaptive immunity, its role is pivotal—upon T cell receptor (TCR) binding to peptide-loaded MHC (pMHC), Arp2/3 is activated by upstream regulators (e.g., WASp) to drive branched actin polymerization at the immunological synapse (IS), which stabilizes the IS,promotes TCR clustering, and recruits key signaling molecules (e.g., Zap70, LAT) to ensure efficient TCR signal initiation/amplification for T cell activation and effector functions (e.g., cytokine secretion), while for B cells, it supports BCR-driven signal activation, B cell proliferation/differentiation into plasma cells, and antibody secretion—all dependent on its modulation of actin dynamics—making Arp2/3 a promising target for immune-related disease therapies,as it regulates actin homeostasis and underpins critical immune steps; despite these findings, key mechanistic gaps remain to advance the framework of “Arp2/3 as a context-dependent immune regulator,” including how Arp2/3 distinguishes immune signals (e.g., “synapse-vs. migration-specific” branching in T cells) and whether post-translational modifications (e.g., Arp3 Ser418 phosphorylation) act as “signal filters,” what defines the functions of Arp2/3 variants (e.g., ARPC1A-vs. ARPC1B-containing complexes) in immune cells and if variant-specific interactors (e.g., ARPC1B-WASP) are the key to functional specificity, how tissue microenvironments (e.g., lymph node stromal factors) buffer Arp2/3 dysfunction (e.g., DC migration discrepancies between *in vitro* and *in vivo*), and whether subtype-specific Arp2/3 defects drive distinct diseases (e.g., ARPC5 for immunodeficiency, ARPC1B for autoimmunity) and what the molecular link between variants and disease subtypes is.
